# Selective glucose sensing in complex media using a biomimetic receptor†

**DOI:** 10.1039/c9sc05406e

**Published:** 2020-02-25

**Authors:** Robert A. Tromans, Soumen K. Samanta, Andy M. Chapman, Anthony P. Davis

**Affiliations:** School of Chemistry, University of Bristol Cantock's Close Bristol BS8 1TS UK Anthony.Davis@bristol.ac.uk; Carbometrics Ltd., Unit DX St Philips Central, Albert Road Bristol BS2 0XJ UK

## Abstract

Glucose is a key biomedical analyte, especially relevant to the management of diabetes. Current methods for glucose determination rely on the enzyme glucose oxidase, requiring specialist instrumentation and suffering from redox-active interferents. In a new approach, a powerful and highly selective achiral glucose receptor is mixed with a sample, l-glucose is added, and the induced CD spectrum is measured. The CD signal results from competition between the enantiomers, and is used to determine the d-glucose content. The involvement of l-glucose doubles the signal range from the CD spectrometer and allows sensitivity to be adjusted over a wide dynamic range. It also negates medium effects, which must be equal for both enantiomers. The method has been demonstrated with human serum, pre-filtered to remove proteins, giving results which closely match the standard biochemical procedures, as well as a cell culture medium and a beer sample containing high (70 mM) and low (0.4 mM) glucose concentrations respectively.

## Abstract 2

A highly selective receptor, circular dichroism and chiral competition are combined in this versatile method for d-glucose analysis.

## Graphical abstract

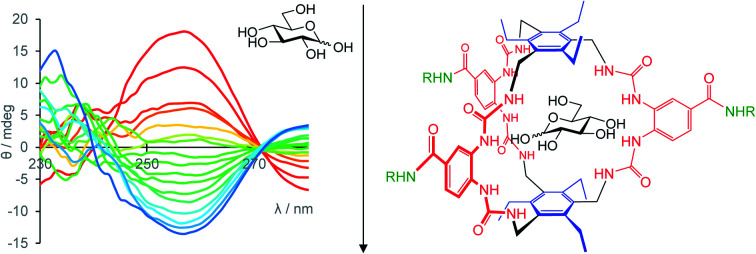

## Introduction

The determination of glucose concentrations is one of the most important analytical problems in chemistry.^1^ More than 400 million people around the world are affected by diabetes. Around 10% are Type 1, who depend on injected insulin and must determine their blood glucose levels several times each day. Type 2 diabetics (the remaining 90%) also require regular blood glucose analyses. There is need both for portable, routine measurement methods and for accurate laboratory-based techniques. Current methodology is based, almost exclusively, on measuring the rate of glucose oxidation catalysed by the enzyme glucose oxidase.^1*d*,*e*^ Although very well-developed, it suffers from interference by redox-active species (*e.g.* ascorbic acid and paracetamol^2^), requires specialist equipment and tends to lose accuracy at low glucose concentrations. Alternatives based on glucose receptors have been intensively studied,^1*a*^ and are beginning to make headway. For example, carbohydrate recognition by boronic acids is well-known,^3^ and has been exploited in an FDA-approved implantable glucose monitor.^4^ However, designing boron-based receptors showing high selectivity for specific substrates remains challenging. Receptors employing non-covalent interactions have been reported by ourselves^5^ and others,^6^ but have tended to show low affinities and interference from non-carbohydrate substrates. The lectin concanavalin A has also been exploited,^7^ but even this natural receptor shows low selectivity for glucose.^8^

**Fig. 1:**
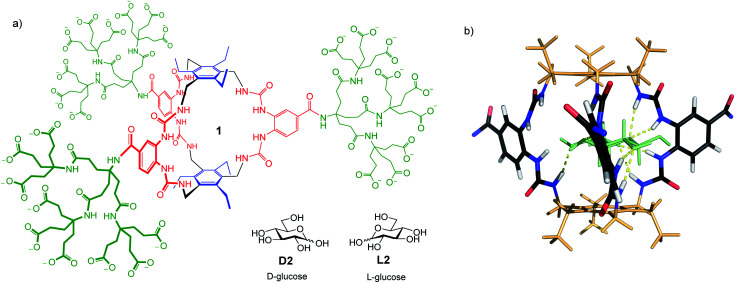
(a) Biomimetic glucose receptor **1**, with d- and l-glucose substrates. (b) Model of **1** binding d-glucose **D2** (shown as pale green). The bis-ureido-benzenecarboxamido chromophores adopt twisted orientations in the complex. Polycarboxylate side chains are omitted for clarity. For details of modelling see ref. 9.

We recently described a glucose receptor **1** (Fig. 1a) with remarkable properties.^9^ The design is based on the previously-studied “temple” architecture,^5^ but features *C*_3_ symmetry and bis-urea “pillars”, preorganised for binding to vicinal OR groups. The affinity of **1** for glucose in water, at 18 000 M^−1^, is ∼100 times higher than any previous designs, and compares well with natural systems. Selectivity for glucose is outstanding. Of all carbohydrates tested, only a few with all-equatorial substitution patterns showed significant binding. Glucose : galactose selectivity, for example, was 130 : 1, while selectivity for glucose *vs.* fructose (often a good substrate for boron-based receptors^1*a*^) was 360 : 1. Binding to a range of other relevant small molecules (*e.g.* amino acids, nucleobases, paracetamol, ascorbic acid^10^) was undetectable. Affinities were unaffected by changes in pH, and only slightly lowered in biological media. Given this performance, receptor **1** has clear potential for application in glucose analysis, provided read-out methodology can be developed. Here we show how **1** can be exploited directly in laboratory-based (as opposed to portable) procedures for glucose determination in complex real-world samples. The methodology requires standard, non-specialist equipment and features adjustable sensitivity, allowing measurements over a wide range of glucose concentrations.

## Results and discussion

The application of **1** to practical glucose sensing required the resolution of two issues. First, we needed a straightforward means of detecting binding, compatible with complex biological media. Second, we had to control sensitivity to allow measurements at relevant concentrations. Here, the high affinities shown by **1** worked against us. Normal blood glucose concentrations are ∼6 mM, while the range of interest for diabetics is from 2 mM (dangerously low) to ≥12 mM (dangerously high). For other applications concentrations may be still higher (for example, up to 1 M for fermentation broths^11^). Receptor **1** is 97% saturated at [glucose] = 2 mM,^12^ so is almost unaffected by concentration changes above this level.

To solve both problems, we turned to circular dichroism (CD). This technique is especially suitable for monitoring carbohydrate recognition because saccharide substrates are chiral but do not contain chromophores. Their influence on receptors, which are usually achiral and light-absorbing, is thus easy to detect.^13^ Receptor **1** contains bis-ureido-benzenecarboxamido units (coloured red in Fig. 1a) which absorb light with *λ*_max_ ∼ 260 nm. As shown in Fig. 1b, binding to glucose was expected to cause twisting of these chromophores, leading to strong CD signals.

**Fig. 2:**
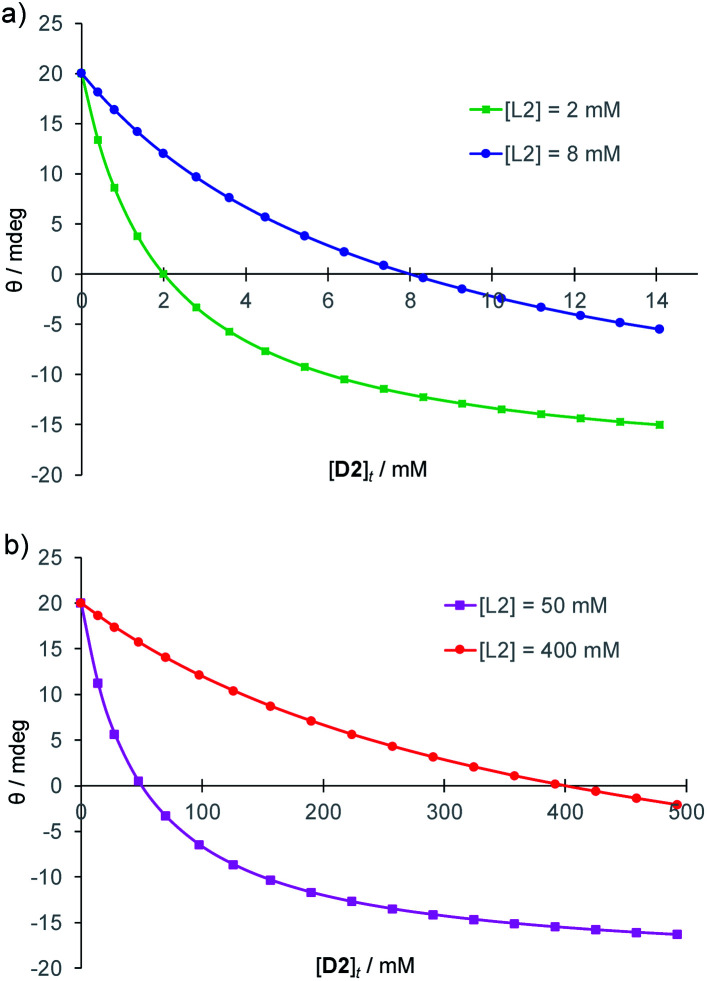
Predicted CD signals *θ* from addition of (chiral) **D2** to achiral, light-absorbing **1** in the presence of **L2** at varying concentrations. Curves are derived from eqn (1), with constant *k* set arbitrarily to 20. (a) Glucose concentrations relevant to blood samples. (b) Higher concentrations relevant to fermentation samples.

A further advantage of CD detection, not previously exploited, is that the response can be tuned, and the signal range expanded, by adding the antipode of the substrate. For example, in the case of **1**, l-glucose **L2** will compete for the binding site with d-glucose **D2** on equal terms. Addition of excess l-glucose will generate the CD spectrum of the complex **1·L2**. Increasing amounts of d-glucose will then reduce the intensity of the spectrum, nullify exactly at [**D2**] = [**L2**], then generate spectra of opposite sign. Because the signal passes from negative to positive, the spread of values is double that given by simple **D2** addition. If the receptor is saturated throughout, a good assumption in the present case, it is readily shown that the CD signal *θ* is given by eqn (1):^14^1
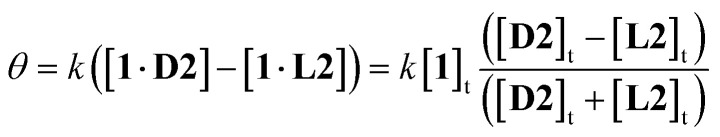
Here *k* is a constant dependent on wavelength, path length, instrument *etc.*, and [**1**]_t_, [**D2**]_t_, and [**L2**]_t_, are total concentrations of receptor, d-glucose and l-glucose in bound and unbound forms. Note that the affinity of **1** for **D2**/**L2** does not appear in the equation. Provided the receptor is saturated the binding constant is irrelevant, so that minor changes due to medium *etc.* are unimportant. Predicted CD output curves for four widely varying [**L2**]_t_ (2, 8, 50 and 400 mM) are shown in Fig. 2. In all cases the slopes are highest for low [d-glucose], but the curves for higher [**L2**]_t_ are clearly more suitable for determinations in the higher concentration ranges (and *vice versa*). Indeed, it can be shown that for a given [**D2**]_t_, the slope ∂*θ*/∂[**D2**]_t_ passes through a maximum at [**D2**]_t_ = [**L2**]_t_ (see ESI†). This provides a simple rule for optimising sensitivity; for maximum response around a certain d-glucose concentration, a similar concentration of l-glucose should be added such that measured *θ* is close to zero. Fig. 2 highlights the wide dynamic range of the method. In principle, measurements can be made at any concentration in the low millimolar range and above.

**Fig. 3:**
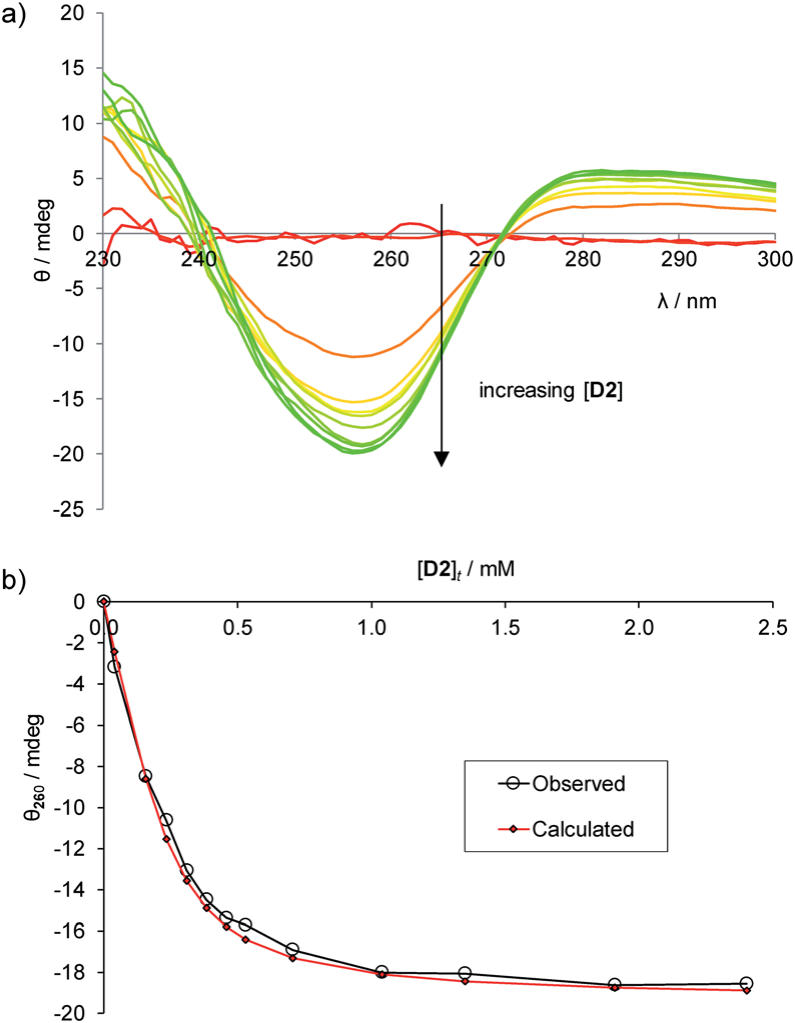
(a) CD spectra from the titration of d-glucose into **1** (0.25 mM) in aqueous phosphate buffer (10 mM, pH = 7.4). Cell path length = 0.1 cm, 16 scans accumulated at each concentration. (b) Curve-fitting analysis of the CD signal at 260 nm, assuming a 1 : 1 binding model. *K*_a_ = 17 200 M^−1^.

To establish the practicality of this approach, we first needed to show that addition of glucose to **1** produced a substantial CD signal. **D2** was titrated into a solution of **1** (0.25 mM) in aqueous phosphate buffer^15^ to give the series of CD spectra shown in Fig. 3a. The changes were monitored at 260 nm and analysed to give a binding constant *K*_a_ of 17 200 M^−1^, consistent with other methods^9^ (Fig. 3b). As expected, addition of **L2** to **1** produced a similar but opposite CD response (Fig. S2†), while titration of **D2** into **1·L2** caused attenuation of the CD spectrum, with reduction to baseline when [**D2**]_t_ = [**L2**]_t_, followed by inversion of the spectrum at higher [**D2**] (Fig. S3†).

**Fig. 4:**
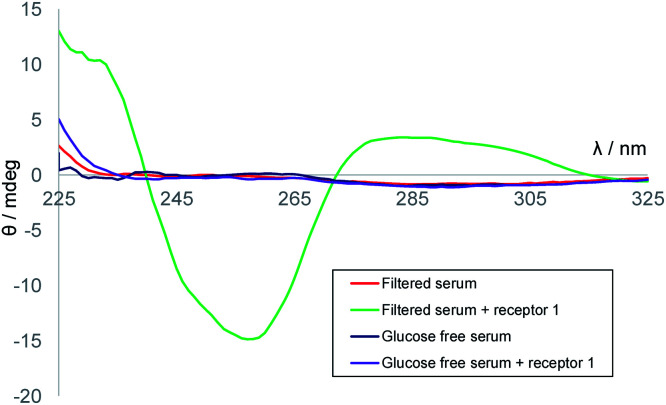
CD spectra of: 10 000 MWCO-filtered human serum (red); filtered serum + receptor **1** (0.25 mM) (green); filtered serum treated with glucose oxidase (glucose-free serum, orange); filtered serum treated with glucose oxidase + receptor **1** (0.25 mM) (purple).

We next considered applying the method to real-world biological samples. As mentioned earlier, we had previously tested receptor **1** against a variety of potential substrates and observed remarkable selectivity for glucose. Interferences from other small molecules were therefore not expected. The affinity of **1** for glucose had been found to be somewhat affected by the medium – for example, in cell culture media containing divalent metal ions, *K*_a_ was reduced to ∼5300 M^−1^. However, as the method involves competition between D and l-glucose such effects need not be important. Even at these lower affinities the receptor should be nearly saturated at relevant glucose concentrations, and deviations from eqn (1) should be small. A further potential issue was background CD absorption from samples of practical interest. Indeed, CD spectra of human serum contained strong signals with sharply irregular structures, presumably due to the high protein content (Fig. S4†). However, filtration through 10 000 molecular weight cut-off (MWCO) membranes, a quick and easy process, gave solutions with negligible CD absorption (see Fig. 4). Addition of receptor **1** to these filtered samples gave the CD spectra expected for **1·D2**, formed from **1** + serum d-glucose. Treatment of serum with glucose oxidase and catalase (to remove glucose), filtration and addition of **1** resulted in a minimal CD signal (Fig. 4). Titration of d-glucose into this solution^15^ then gave spectra, similar to those in Fig. 3a, which were analysed to give *K*_a_ = 10 300 M^−1^, consistent with earlier ITC measurements performed in similar media (Fig. S6†).

With these results in hand, we were positioned to develop methods to measure d-glucose levels in serum. To aid sample manipulation and ensure consistency, the serum was routinely diluted by 50% with 20 mM phosphate buffer (pH 7.4) containing **1** and l-glucose as necessary.^15^ Measured d-glucose levels were then doubled to give the values in the original sample.

In a first experiment, receptor **1** was added to filtered diluted serum, still containing the endogenous d-glucose, and the CD spectrum was monitored while serum containing l-glucose was titrated into the solution.^15^ The CD signal was found to pass through zero for [**L2**] in the range 2.85–2.95 mM, equivalent to 5.7–5.9 mM in the serum (Fig. S7†). For comparison, the serum d-glucose concentration was also measured using an enzyme-based YSI analyser, and found to be 5.8 mM ± 0.1 mM. The YSI instrument is the standard equipment for laboratory glucose analysis and commonly used as a reference in studies of blood glucose monitors.^16^

**Fig. 5:**
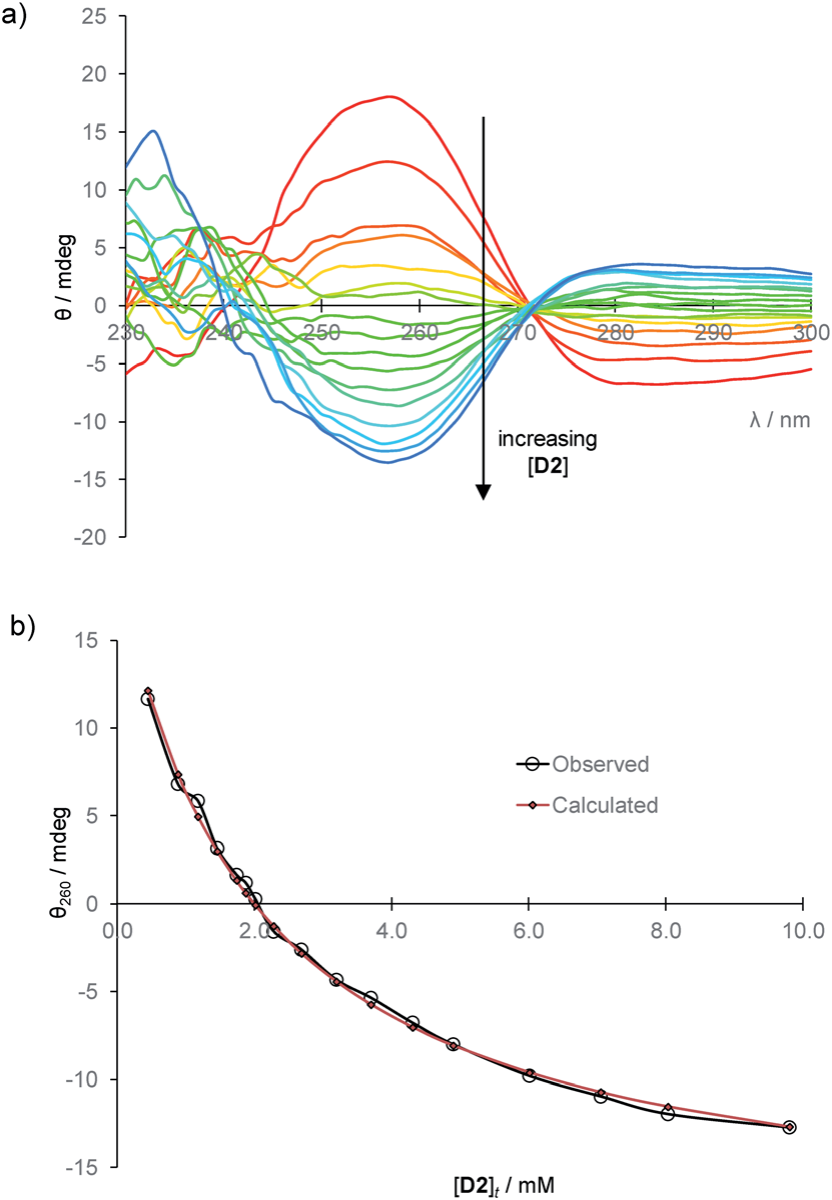
(a) CD spectra from the titration of d-glucose into filtered, diluted, glucose-free human serum, in the presence of l-glucose (2 mM) and **1** (0.25 mM). (b) Curve-fitting of the CD signal at 260 nm to eqn (1) with variation of constant *k*. The best fit (red diamonds) was obtained for *k* = −76 800 mdeg M^−1^.

To establish a more convenient procedure, we performed calibration experiments as follows. Glucose-free serum was prepared by the glucose oxidase/catalase method described above. l-Glucose (2 mM or 8 mM) and receptor **1** (0.25 mM) were added, with dilution, to give a titrand. A titrant containing the same concentrations of **L2** and **1**, with the addition of d-glucose (40 mM), was prepared and added to the titrand. CD spectra were acquired, corresponding to [**D2**] up to ∼10 mM at constant concentrations of **1** and **L2**. The spectra obtained for [**L2**] = 2 mM are shown in Fig. 5a. This set of data provides an empirical calibration curve allowing measurement of [**D2**] from single CD spectra using the given spectrometer and settings, [**L2**] and [**1**].

More usefully, it was found that the variations in CD absorption at 260 nm (*θ*_260_) gave an excellent fit to eqn (1) if *k* was allowed to vary (Fig. 5b). This confirmed the validity of eqn (1),^17^ while also providing an accurate value of *k*. Once *k* has been determined, it is thus possible to obtain [**D2**] from a single measurement of *θ*_260_ simply by applying eqn (2), obtained by rearrangement of eqn (1) (see ESI†).2
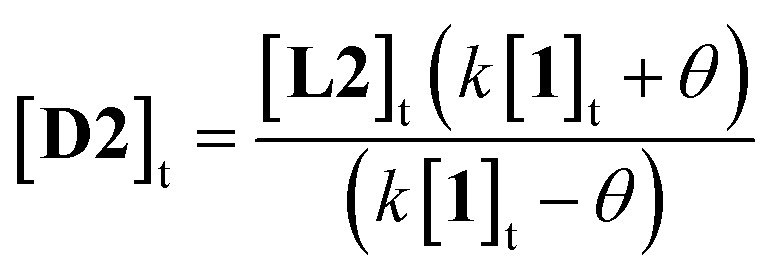


The optimised protocol thus involves (a) centrifugal filtration of the sample through a 10k MWCO membrane, (b) dilution by 50% with a standard solution of **1** and **L2** in phosphate buffer, adjusted to give the concentrations used for the calibration experiment, (c) measurement of the CD signal at 260 nm, and (d) application of eqn (2), doubling the initial answer to account for the dilution. The procedure was applied to the filtered serum in six independent experiments, three employing [**L2**] = 2 mM and three with [**L2**] = 8 mM. Results varied from 5.58 to 5.87 mM, with an average of 5.75. Further experiments would be needed to optimise the procedure and define the accuracy more precisely, but these initial results suggest a similar performance to the industry-standard YSI analyser.

To demonstrate the versatility of the method we performed analyses in two further media. Firstly we employed a cell culture medium, which would typically contain quite high glucose concentrations requiring correspondingly high [**L2**].^11^ The medium was obtained glucose-free, receptor **1** was added, and a calibration curve was measured in the presence of l-glucose (50 mM) as described above for the glucose-free serum. Addition of d-glucose caused the expected changes to the CD spectrum, which again fitted well to eqn (1) (Fig. S10†). A sample for analysis was then prepared by adding d-glucose (70 mM) to the cell culture medium and subjected to the standard protocol with [**L2**] = 50 mM (see ESI†). The results were within 2% of the expected value (Table S5†). Secondly, to illustrate the application to samples with lower [**D2**], we employed beer with an artificially reduced glucose concentration.^18^ As described in the ESI,† the d-glucose was first removed enzymatically from the beer, and a calibration curve was measured in the presence of 0.2 mM l-glucose. In this case eqn (1) did not apply, as expected for such a low [**L2**], but the data could be fitted to an empirical equation in Excel (Fig. S11†). The sample for analysis was generated by addition of d-glucose (0.4 mM) to the glucose-free beer, and subjected to the standard protocol with [**L2**] = 0.2 mM. d-Glucose concentrations could be obtained by solving the empirical equation. Again, the results were within 2% of those expected (Table S6†).

## Conclusions

In conclusion, we have shown that hexaurea receptor **1** can be applied to the accurate analysis of d-glucose concentrations in complex biological mixtures. The extreme selectivity observed for **1** implies that interferences will be negligible. Unusually, the sensitivity is adjustable so that samples of widely different concentrations can be handled with ease. In terms of equipment, the procedure requires only a centrifuge and a CD spectrometer, multi-purpose instruments that are available in many (bio)chemical laboratories. The technique could thus complement the standard methods based on glucose oxidase, for cases where redox-active species can cause interference and/or specialist instrumentation may not be available.

## Conflicts of interest

There are no conflicts to declare.

## Supplementary Material

SC-011-C9SC05406E-s001
