# Supplementary material for: Selective glucose sensing in complex media using a biomimetic receptor
Source: Chem Sci. 2020 Feb 25;11(12):3223–7. doi: 10.1039/c9sc05406e (PMC8157503; doi:10.1039/c9sc05406e)
Supplement: SC-011-C9SC05406E-s001 [file SC-011-C9SC05406E-s001.pdf]

Electronic Supplementary Material (ESI) for Chemical Science.
This journal is © The Royal Society of Chemistry 2020

Selective Glucose Sensing in Complex Media using a Biomimetic Receptor

Robert A. Tromans,[1] Soumen K. Samanta,[1] Andy M. Chapman[2] and Anthony P. Davis[1*]

[1]School of Chemistry, University of Bristol, Cantock's Close, Bristol, BS8 1TS, UK.

[2]Carbometrics Ltd., Unit DX, St Philips Central, Albert Road, Bristol BS2 0XJ, UK

Table of Contents

Methods 2

CD Binding studies 2

Preparation of biological media. 3

Theoretical background 4

Binding Studies 7

Titration of Receptor **1** with Glucose in Water 7

Competitive Titration of **1** + L-Glucose with D-Glucose in Water 9

CD Analysis of Human Serum 11

Titration of **1** with D-Glucose in Glucose-Free Human Serum 13

Quantification of D-Glucose in complex media using Receptor 1 14

Overview 14

Titration of Receptor **1** with L-Glucose in Filtered Human Serum 15

Titrations of D-Glucose into Receptor **1** + L-Glucose in Glucose-Free Human Serum (Calibrations for Single-Point Determinations). 16

Single-Point Determination of D-Glucose in Filtered Human Serum 18

Titration of D-Glucose into Receptor **1** + L-Glucose in Glucose-Free Cell Culture Medium (Calibration for Single-Point Determinations). 20

Single-Point Determination of D-Glucose in Glucose-Free Cell Culture Medium 21

Titration of D-Glucose into Receptor **1** + L-Glucose in Glucose-Free Peroni Beer (Calibration for Single-Point Determinations). 22

Single-Point Determination of D-Glucose in Peroni Beer 23

Methods

CD Binding studies

CD titrations were performed on a JASCO J-810 spectropolarimeter or a JASCO J-815 CD spectrometer. In all cases titrant and titrand solutions were identical except for the glucose substrate added to the titrand. Thus, for example, all titrant solutions contained receptor **1** at the concentration used for the experiment. In consequence, only substrate concentrations (**D2** or **L2**, depending on the experiment) changed during the titrations. Solutions prepared from solid glucose were allowed to equilibrate overnight before use. For studies in simple aqueous media, solutions were prepared from HPLC grade water with addition of phosphate buffer (10 mM, pH 7.4). The preparation of biological media is described separately below. Receptor concentrations were typically 100 µM – 250 µM. The receptor solution (250 µL) was placed in a cuvette and aliquots of titrant solution were added. The cuvette was shaken after each addition, allowed to settle for 30 seconds and the CD spectrum acquired at 298 K. Acquisition parameters for the CD spectropolarimeter were typically as follows:

Data pitch	0.5 nm
Data points	151
Bandwidth	1 nm
Response	1 second
Scanning speed	100-200 nm/min
Accumulation	16-24
Cell length	0.1 cm
Temp	298 K
Scan range	225-300 nm

In the absence of a competing guest (e.g. added L-glucose), binding constants were obtained by fitting the induced ellipticity at 260 nm, ϑ_{260}, to a 1:1 binding model using a non-linear least squares curve-fitting programme implemented within Excel. The programme yields binding constants K_a and limiting ϑ_{260} as output. Estimated errors for K_a were obtained from individual data points by assuming the determined K_a and limiting ϑ_{260}.

Preparation of biological media.

Human serum

The human serum used originates from male AB clotted whole blood (cat. no. H6914) and was acquired from Sigma-Aldrich. *Filtered serum* was prepared by centrifugal filtration through a size-excluding membrane (10k MWCO) and then diluted with a phosphate buffer solution (pH = 7.4, 20 or 100 mM) such that the final concentration of added buffer was 10 mM. Species to be added to the medium were introduced during the dilution process.[*] The final pH of the samples was adjusted to 7.4 by addition of aqueous sodium hydroxide or hydrochloric acid as necessary.

The concentration of initial D-glucose present in the human serum was measured to be 5.80 $\pm$0.1 mM using a YSI 2300 STAT Plus Glucose and L-Lactate analyser. *Glucose-free human serum* was prepared by oxidising the D-glucose to D-glucono-δ-lactone (which then hydrolysed to gluconic acid in solution at pH 7.4) using a combination of enzymes glucose oxidase and catalase, as follows. A solution of enzymes (20 mL) consisting of glucose oxidase (10,000 U) and catalase (300,000 U) was prepared and then dialysed (500 MWCO) to remove low MW contaminants (notably glycerol). 0.2 mL of this enzyme solution was added to human serum (10 mL) at 25-30 °C. The mixture was stirred at 25-30 °C while air was bubbled through the suspension for 2 hours. The mixture was then cooled to room temperature and passed through a membrane (10k MWCO) to give a colourless solution, which was diluted with phosphate buffer (to 10 mM added phosphate) and adjusted to pH 7.4 as described above. The samples were then used immediately for the corresponding binding studies. After oxidation, the concentration of D-glucose in the human serum was measured to be 0-0.005 mM using the YSI 2300 STAT Plus.

[*] Dilution of the biological media provides an opportunity to add substances (**1**, **D2**, **L2**) as solutions. This is far more convenient and accurate than the addition of solids, and is the main purpose of adding a dilution step to the procedure.

Glucose-free cell culture medium

Cell culture medium with no D-glucose (SILAC DMEM Flex Media, catalog number : A24939-01) was purchased from Life Technologies Ltd.

Glucose-free Peroni beer

A bottle of Peroni gluten-free beer was obtained, opened, and left to stand overnight. D-Glucose was removed by oxidation to D-glucono-δ-lactone (which then hydrolysed to gluconic acid in solution at pH 7.4) using a combination of enzymes glucose oxidase and catalase, as follows. A solution of enzymes (20 mL) consisting of glucose oxidase (10,000 U) and catalase (300,000 U) was prepared and then dialysed (500 MWCO) to remove low MW contaminants. 0.4 mL of this enzyme solution was added to Peroni beer (10 mL) at 25-30 °C. The mixture was stirred at 25-30 °C while air was bubbled through the suspension for 4 h. The mixture was then cooled to room temperature and passed through a membrane (10k MWCO) to give a pale coloured solution, which was diluted with phosphate buffer (to 10 mM added phosphate) and adjusted to pH 7.4 as described above. The samples were then used immediately for the binding studies.

Theoretical background

As described in the main paper, our method for determining D-glucose concentrations is based on equation (1), where $[\mathbf{1}]_t$, $[\mathbf{D2}]_t$, and $[\mathbf{L2}]_t$, are total concentrations of receptor, D-glucose and L-glucose in bound and unbound forms.

$$\theta = k([\mathbf{1}\cdot\mathbf{D2}] - [\mathbf{1}\cdot\mathbf{L2}]) = k[\mathbf{1}]_t \frac{([\mathbf{D2}]_t - [\mathbf{L2}]_t)}{([\mathbf{D2}]_t + [\mathbf{L2}]_t)} \qquad (1)$$

This relation may be derived as shown below, employing the simpler and more general notation H = host, D = D-glucose, L = L-glucose. To simplify the analysis, we make the assumption that the host is fully saturated at all relevant substrate concentrations. In justification, for K_a = 18,000 M^{-1}, the binding site is 97.3% occupied for [L-glucose] = 2 mM, and 98.6% occupied after an equal amount of D-glucose has been added.

Given the assumption of host saturation, so that [H] ~0, the only equilibrium of significance is:

$$\mathrm{HL} + \mathrm{D} \overset{K_{exch}}{\rightleftharpoons} \mathrm{HD} + \mathrm{L}$$

for which $K_{\mathrm{exch}} = \frac{[\mathrm{HD}][\mathrm{L}]}{[\mathrm{HL}][\mathrm{D}]}$

The binding constants for the host binding D and L glucose are given by:

$$K_{\mathrm{HD}} = \frac{[\mathrm{HD}]}{[\mathrm{H}][\mathrm{D}]} \quad \text{and} \quad K_{\mathrm{HL}} = \frac{[\mathrm{HL}]}{[\mathrm{H}][\mathrm{L}]}$$

and are equal, so that

$$K_{exch} = \frac{[\mathrm{HD}][\mathrm{L}]}{[\mathrm{HL}][\mathrm{D}]} = \frac{K_{\mathrm{HD}}}{K_{\mathrm{HL}}} = 1 \qquad \text{(S1)}$$

Assuming that [H] = 0, the following mass balance equations apply:

$$[\mathrm{H}]_t = [\mathrm{HD}] + [\mathrm{HL}]$$

$$[\mathrm{D}]_t = [\mathrm{D}] + [\mathrm{HD}]$$

$$[\mathrm{L}]_t = [\mathrm{L}] + [\mathrm{HL}]$$

Equation (S1) may now be expressed in terms of [HD], $[\mathrm{H}]_t$, $[\mathrm{L}]_t$, and $[\mathrm{D}]_t$ as follows:

$$K_{exch} = \frac{[\mathrm{HD}]\{[\mathrm{L}]_t + [\mathrm{HD}] - [\mathrm{H}]_t\}}{([\mathrm{H}]_t - [\mathrm{HD}])([\mathrm{D}]_t - [\mathrm{HD}])} = 1$$

Rearranging:

$$[\mathrm{HD}]\{[\mathrm{L}]_t + [\mathrm{HD}] - [\mathrm{H}]_t\} = ([\mathrm{H}]_t - [\mathrm{HD}])([\mathrm{D}]_t - [\mathrm{HD}])$$

Multiplying out:

$$[\mathrm{HD}][\mathrm{L}]_t + [\mathrm{HD}]^2 - [\mathrm{HD}][\mathrm{H}]_t = [\mathrm{H}]_t[\mathrm{D}]_t - [\mathrm{H}]_t[\mathrm{HD}] - [\mathrm{HD}][\mathrm{D}]_t + [\mathrm{HD}]^2$$

Cancelling

$$[\mathrm{HD}][\mathrm{L}]_t = [\mathrm{H}]_t[\mathrm{D}]_t - [\mathrm{HD}][\mathrm{D}]_t$$

From which

$$[\mathrm{HD}] = \frac{[\mathrm{H}]_t[\mathrm{D}]_t}{([\mathrm{L}]_t + [\mathrm{D}]_t)}$$

Similarly

$$[\mathrm{HL}] = \frac{[\mathrm{H}]_t[\mathrm{L}]_t}{([\mathrm{L}]_t + [\mathrm{D}]_t)}$$

The CD signal will be proportional to [HD] – [HL], so:

$$\theta = k([\mathrm{HD}] - [\mathrm{HL}]) = k[\mathrm{H}]_t \frac{([\mathrm{D}]_t - [\mathrm{L}]_t)}{([\mathrm{L}]_t + [\mathrm{D}]_t)} \qquad \text{(S2)}$$

Equation (S2) is equivalent to Equation (1) from the main paper.

Equation (S2) may be differentiated† with respect to $[\mathrm{D}]_t$ to give:

$$\frac{d\theta}{d[\mathrm{D}]_t} = \frac{2k[\mathrm{H}]_t[\mathrm{L}]_t}{([\mathrm{L}]_t + [\mathrm{D}]_t)^2}$$

This function effectively represents the sensitivity of ϑ to changes in $[\mathrm{D}]_t$, for a given amount of added L-glucose. A second differentiation with respect to $[\mathrm{L}]_t$ gives:

$$\frac{\partial^2\theta}{\partial[\mathrm{D}]_t\,\partial[\mathrm{L}]_t} = \frac{2k[\mathrm{H}]_t([\mathrm{L}]_t - [\mathrm{D}]_t)}{([\mathrm{L}]_t + [\mathrm{D}]_t)^3}$$

This function passes through zero when $[\mathrm{L}]_t = [\mathrm{D}]_t$, implying that the first derivative passes through a maximum at that point. For a given value of $[\mathrm{D}]_t$, the slope $\partial\vartheta/\partial[\mathrm{D}]_t$ is therefore greatest when the same amount of L-glucose is added.

† https://www.derivative-calculator.net/

Binding Studies

Titration of Receptor 1 with Glucose in Water

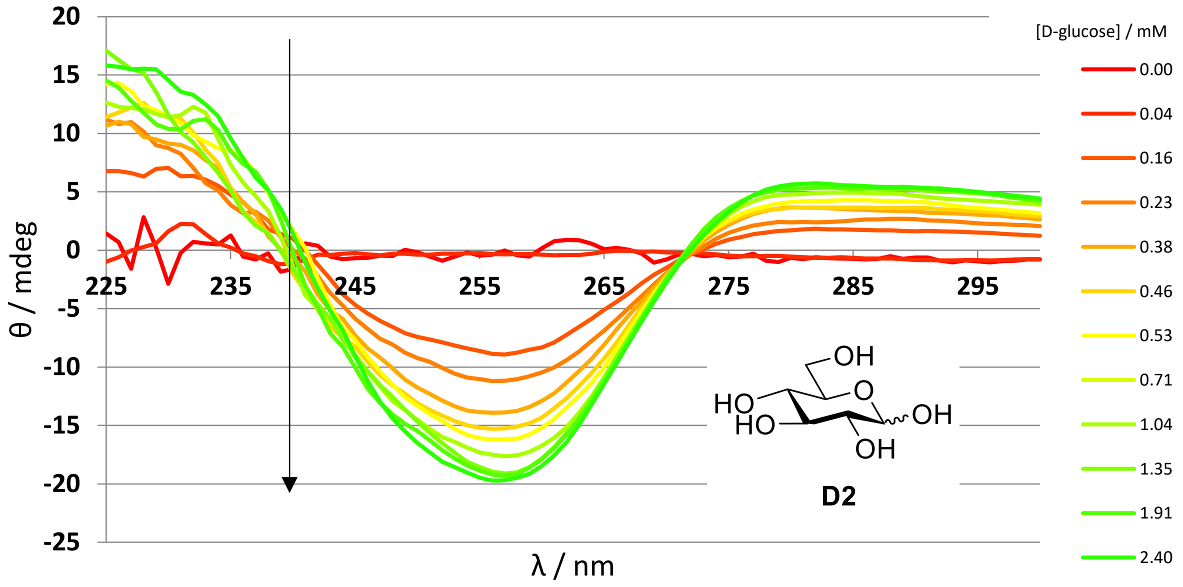

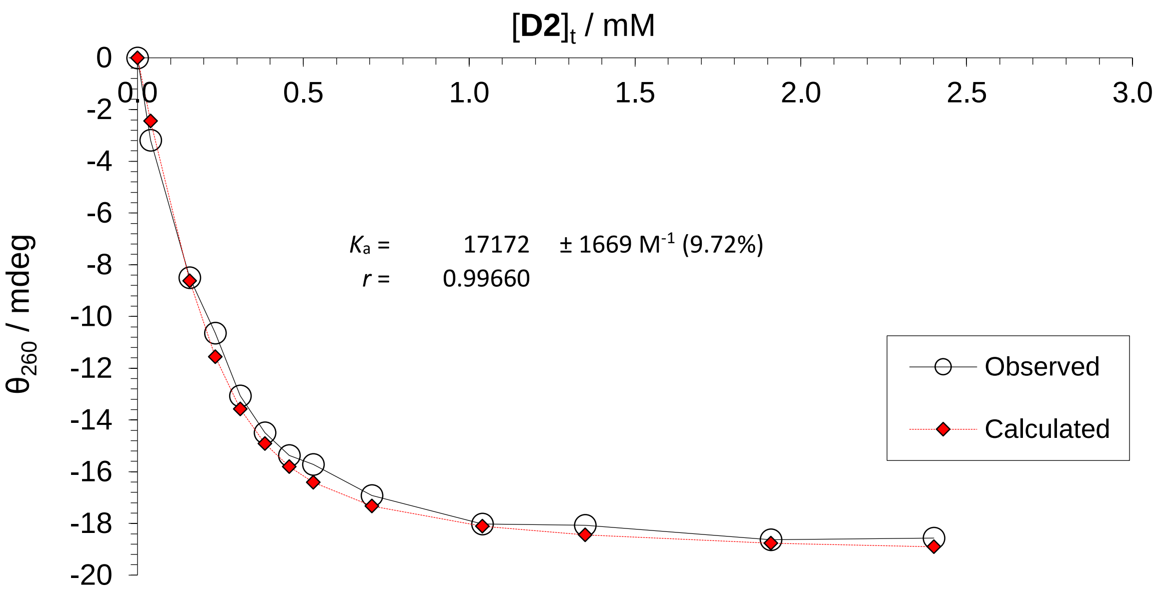

Figure S1 CD spectra (top) and binding analysis curve (bottom) for the titration of D-glucose **D2** (10 mM) into receptor **1** (0.25 mM) in aqueous phosphate buffer (10 mM, pH 7.4) at 298 K. The CD intensity at 260 nm (ϑ_{260}) plotted against increasing guest concentration (mM) and analysed according to a 1:1 binding model. K_a = 17,200 ± 1700 M^{-1} (9.7%).

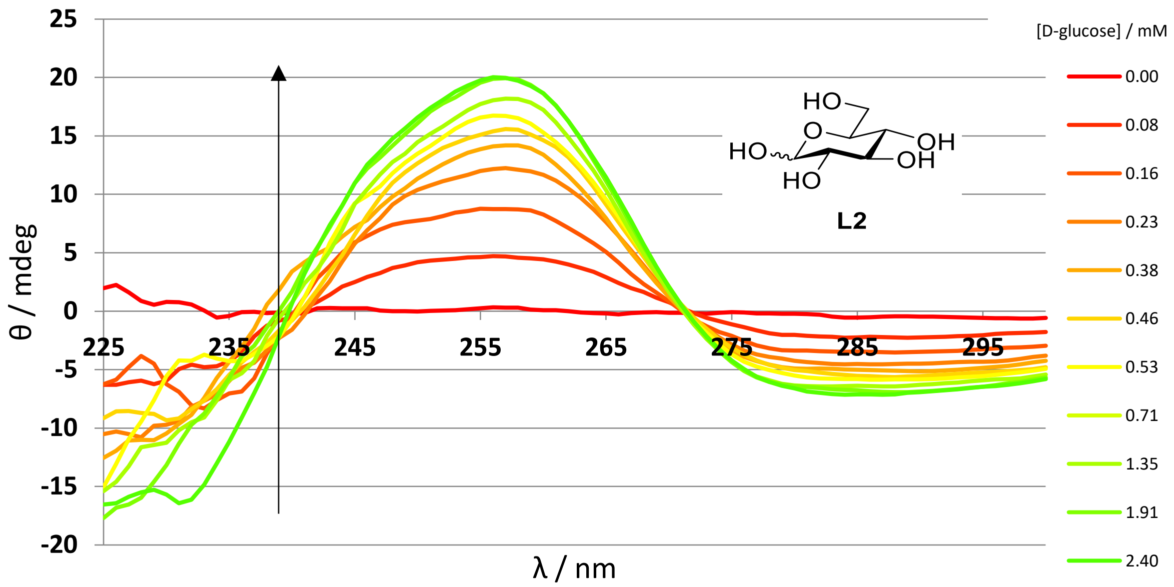

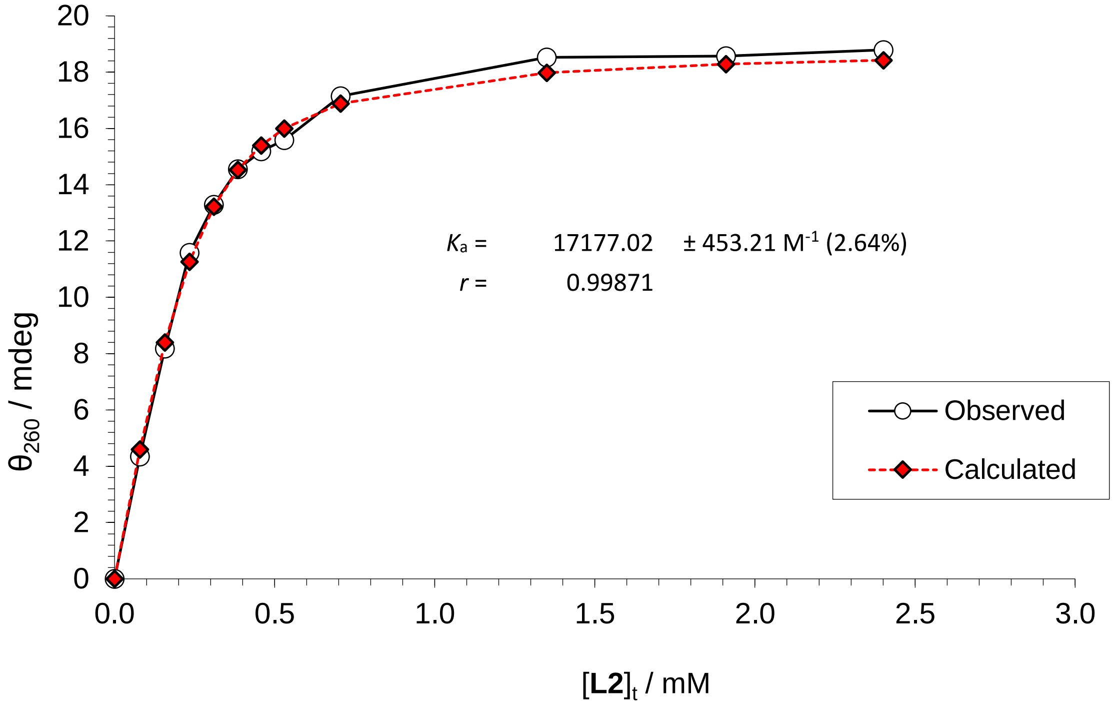

Figure S2 CD spectra (top) and binding analysis curve (bottom) for the titration of L-glucose **L2** (10 mM) into receptor **1** (0.25 mM) in aqueous phosphate buffer (10 mM, pH 7.4) at 298 K. The CD intensity at 260 nm (ϑ_{260}) plotted against increasing guest concentration (mM) and analysed according to a 1:1 binding model. K_a = 17,200 ± 360 M^{-1} (2.1%).

Competitive Titration of 1 + L-Glucose with D-Glucose in Water

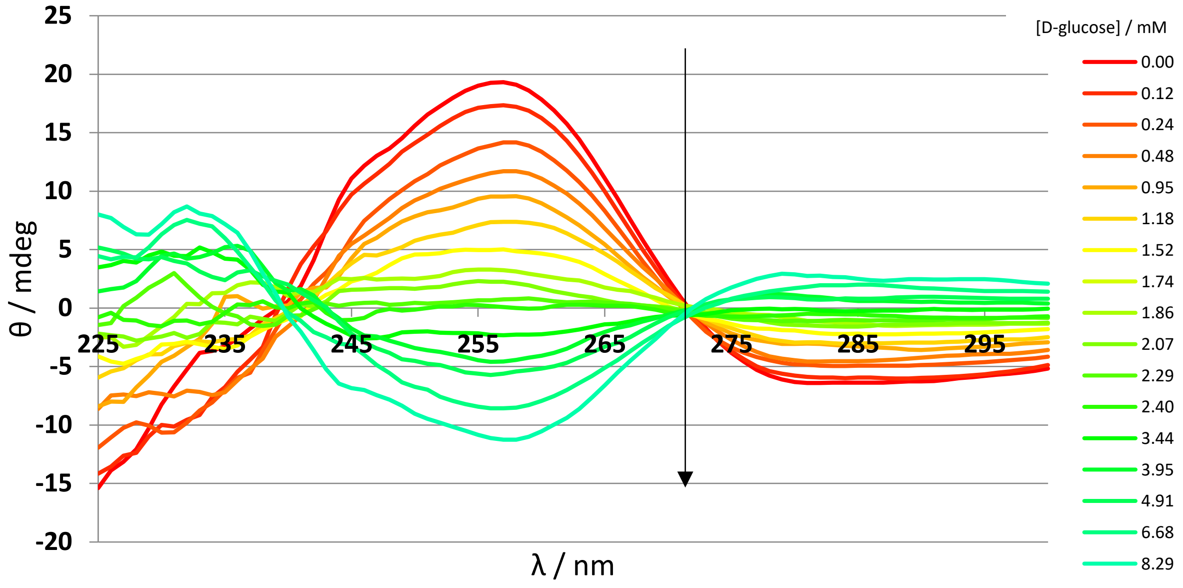

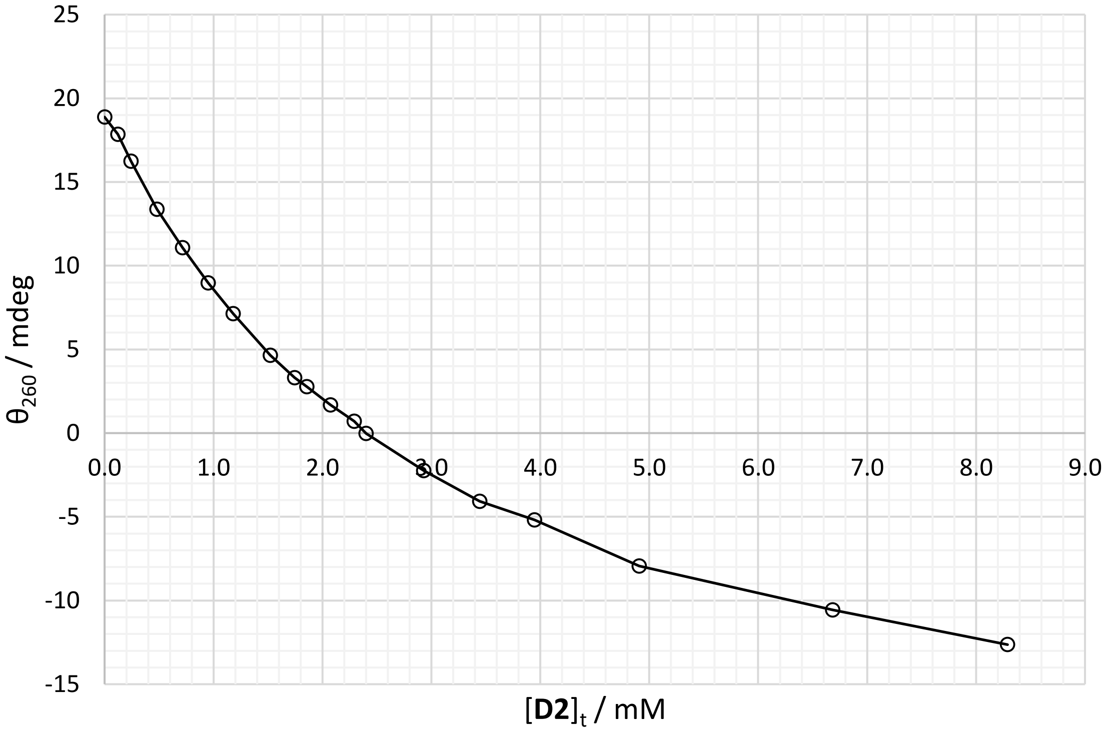

Figure S3 Top: CD spectra for the titration of D-glucose **D2** (10 mM) into receptor **1** (0.25 mM) plus L-glucose **L2** (2.4 mM) in aqueous phosphate buffer (10 mM, pH 7.4) at 298 K. Bottom: The CD intensity at 260 nm (ϑ_{260}) plotted against increasing concentration of **D2** (mM). Zero signal is observed when ratio of [D-glucose]:[L-glucose] is equal to one (see also Table S1).

Table S1 Tabulated data from the experiment outlined in Figure S3. The CD signal passes through zero when ratio of D-glucose **D2** and L-glucose **L2** concentrations are equal (highlighted in blue).

[D2] / mM	ϑ_{260} / mdeg
0.00	18.88
0.12	17.85
0.24	16.25
0.48	13.37
0.72	11.08
0.95	8.97
1.18	7.13
1.52	4.65
1.74	3.31
1.86	2.78
2.07	1.68
2.29	0.71
2.40	-0.02
2.93	-2.24
3.44	-4.07
3.95	-5.18
4.91	-7.94
6.68	-10.55
8.29	-12.64

CD Analysis of Human Serum

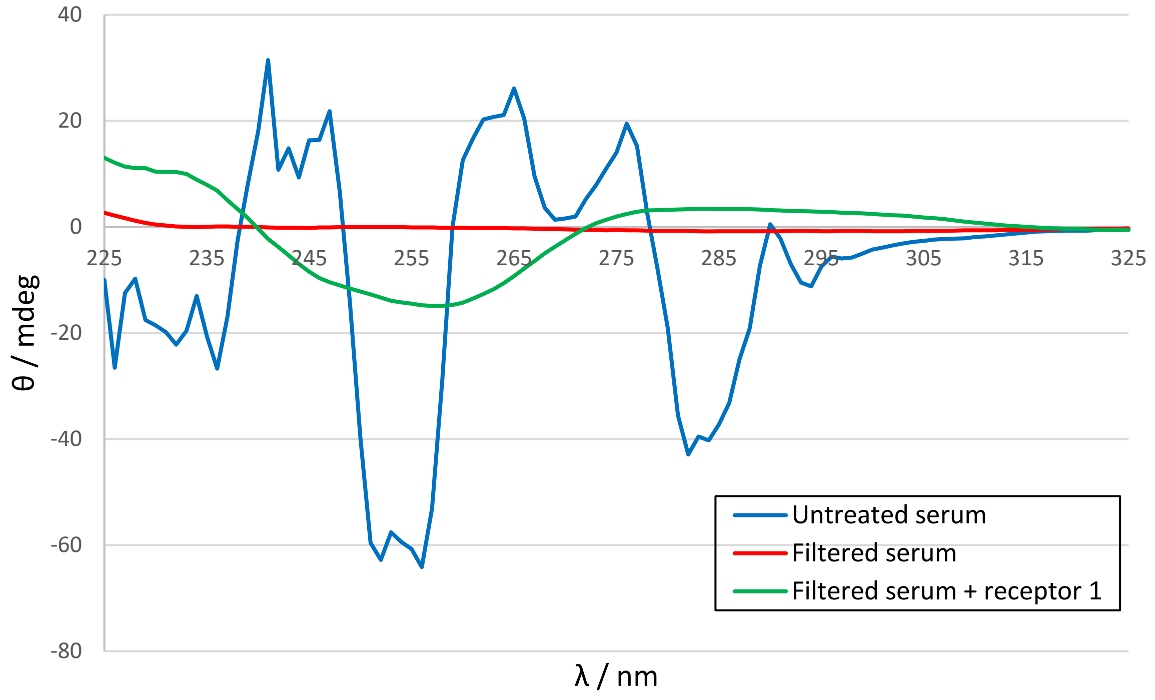

Figure S4 *Untreated serum, filtered serum, and addition of receptor **1***. The blue trace represents raw unfiltered serum that had been diluted by 10% with 0.1 M phosphate buffer solution then adjusted to pH 7.4. This unfiltered serum produced very large absorption artefacts, presumably due to high molecular weight components (proteins, antibodies etc.) or light scattering from insoluble particles. The red trace represents human serum filtered through a membrane (10k MWCO) then diluted by 10% with 0.1 M phosphate buffer solution then adjusted to pH 7.4. This filtered serum medium gave little to no observable CD absorption. Addition of receptor (final receptor concentration of 0.25 mM) to this filtered serum medium induced a CD signal (green line), due to the binding of **1** to D-glucose **D2**. All spectra acquired at 298 K.

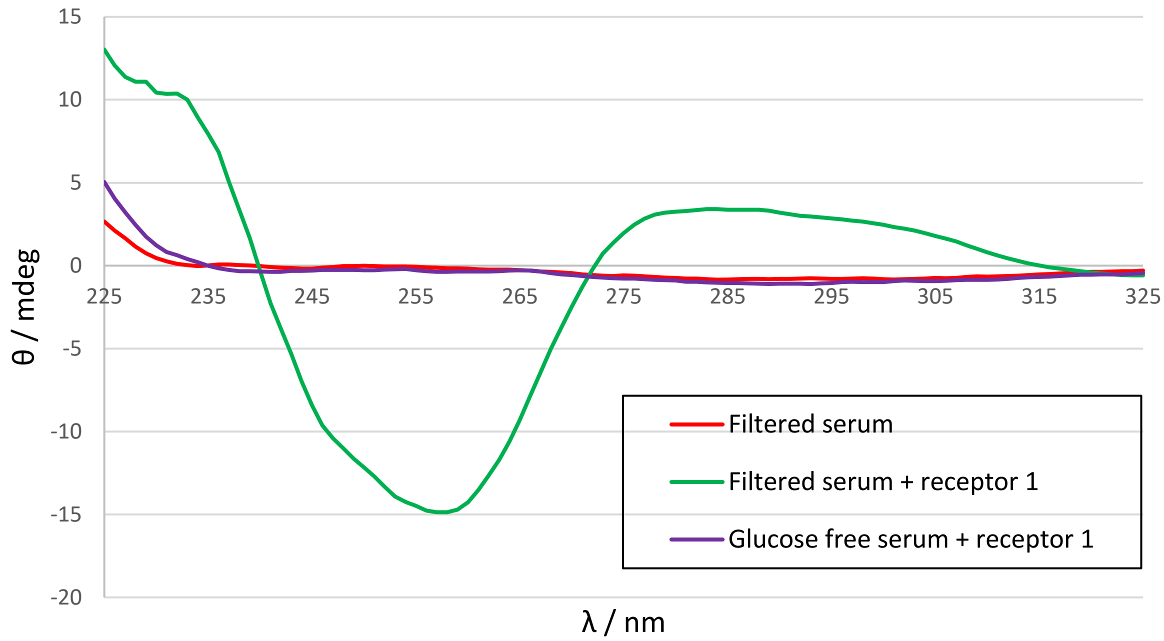

Figure S5 *Effect of D-glucose removal.* The red and green traces represent filtered human serum, and filtered human serum + receptor **1**, as in Fig. S3 above. The purple trace represents glucose-free human serum, diluted by 10% with 0.1 M phosphate buffer solution then adjusted to pH 7.4, to which receptor **1** (0.25 mM) has been added. The absence of significant CD absorption confirms that the glucose has been removed by oxidation and that the products do not produce a CD signal in the presence of **1**.

Titration of 1 with D-Glucose in Glucose-Free Human Serum

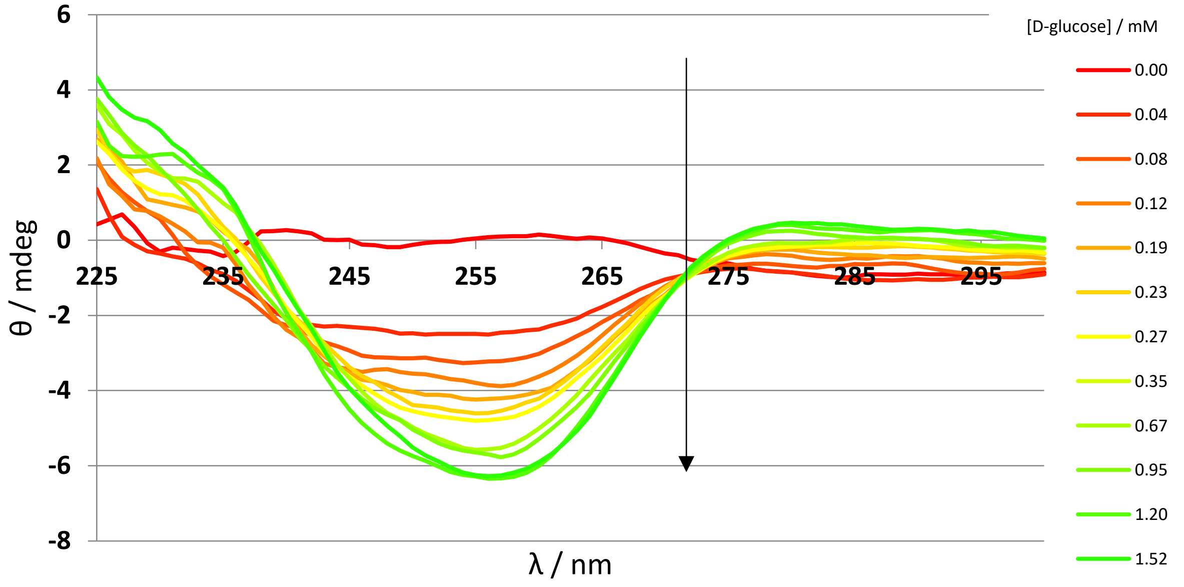

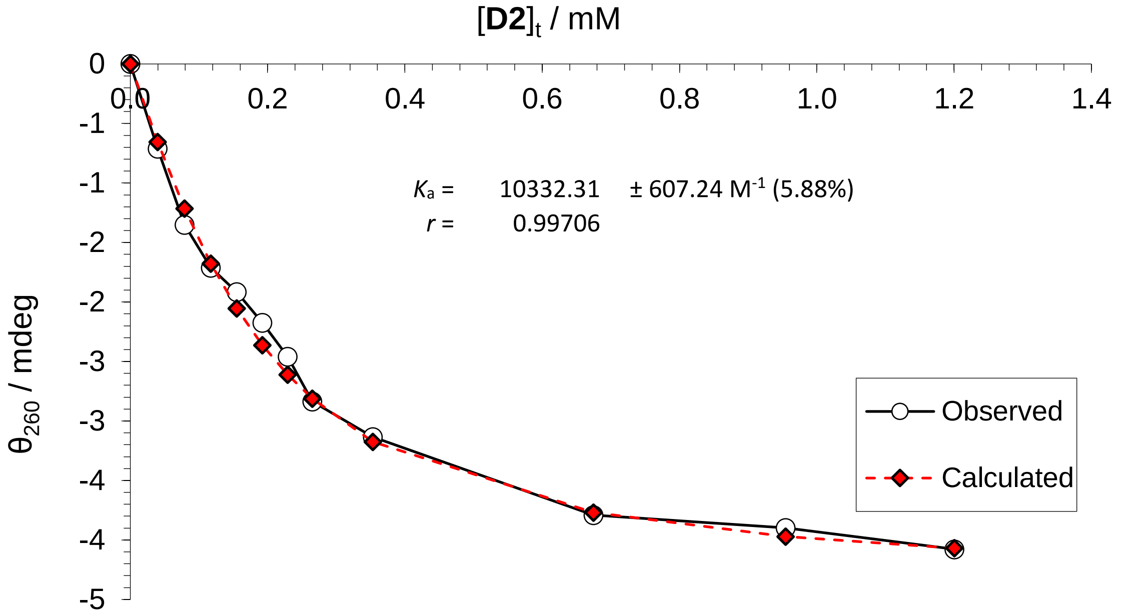

Figure S6 CD spectra (top) and binding analysis curve (bottom) for the titration of D-glucose **D2** (5 mM) into receptor **1** (0.15 mM) in glucose-free human serum. The serum was diluted by 10% with 0.1 M phosphate buffer solution then adjusted to pH 7.4 before use. The CD intensity at 260 nm (ϑ_{260}) was plotted against increasing guest concentration (mM) and analysed according to a 1:1 binding model. K_a = 10,300 ± 610 M^{-1} (5.9%).

Quantification of D-Glucose in complex media using Receptor 1

Overview

In all the following experiments, biological media prepared as described earlier were diluted by 50% with phosphate buffer (20 mM), with addition of other components as necessary. Titrant solutions were prepared by incorporating glucose (**L2** or **D2**) in titrand solutions, such that only glucose concentrations changed during the titrations.

Figure S7 and Table S2 show the results from titration of **L2** into receptor **1** in filtered human serum which still contains endogenous **D2**. This approach provides a reliable method for the determination of D-glucose in filtered human serum, but is time-consuming.

Figures S8 and S9 show the results from the titration of D-glucose into glucose-free human serum, to which receptor **1** and L-glucose (2 mM or 8 mM) has been added. The experimental data could be used as an empirical calibration curves to allow single-step measurements of unknown glucose concentrations in the same media. Alternatively, as shown in the Figures, the curves fit well to equation (1) (or S2, which is equivalent) if *k* is allowed to vary, providing a simple method for extracting the glucose concentration from the data. Repeated application of the method to the serum give highly consistent results as shown in Tables S3 and S4.

Figure S10 and Table S5 give the results from applying a similar procedure to analysis of relatively high levels of D-glucose in a cell culture medium. Figure S10 shows the calibration curve obtained from titrating D-glucose into the (D-glucose-free) medium, in the presence of 50 mM L-glucose. Again the data fits well to equation (1), which can then be applied to the single-point measurements. The sample for analysis was prepared by adding D-glucose (70 mM) to the medium.

Figure S11 and Table S6 show the results from applying the method to relatively low levels of D-glucose in beer. Preliminary tests on the beer chosen (Peroni) showed that the D-glucose concentration, at ~4 mM, was higher than we required. After removing the glucose for the calibration curve, we therefore added 0.4 mM D-glucose to obtain the sample for analysis. The calibration curve was obtained in the presence of 0.2 mM L-glucose, too low for the application of equation (1). Instead the curve was fitted to an empirical polynomial equation, which was solved to give the single-point measurements in Table S6.

Titration of Receptor 1 with L-Glucose in Filtered Human Serum

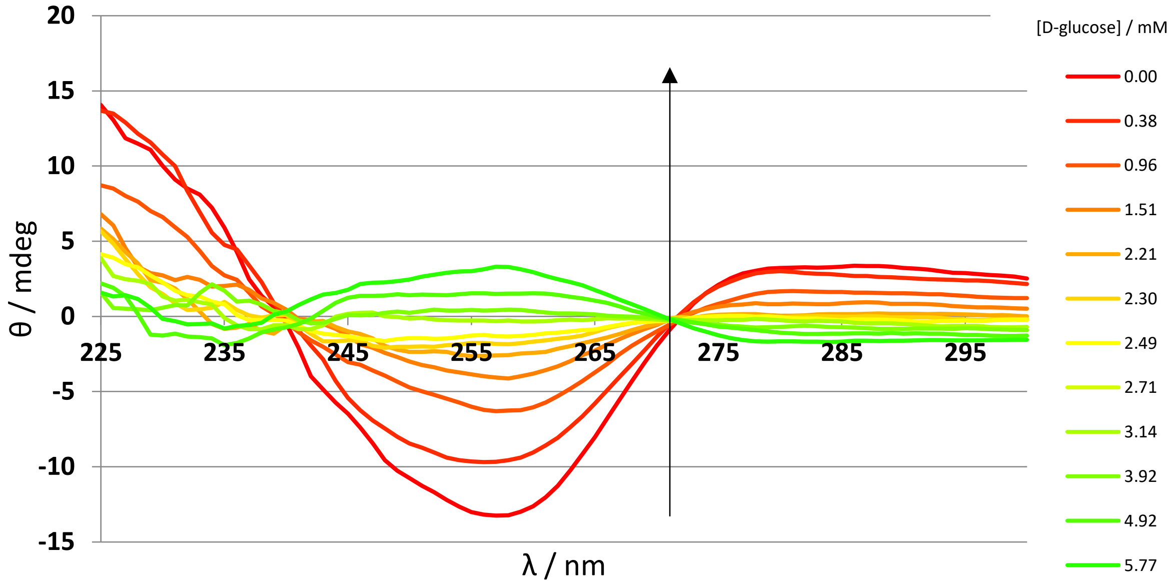

Figure S7 CD spectra for the titration of L-glucose **L2** (20 mM) into receptor **1** (0.25 mM) in filtered diluted human serum (50% v/v) at 298 K.

Table S2 CD intensities at 260 nm (ϑ_{260}) from the experiment represented in Figure S7. The CD signal is close to zero when the ratio of D-glucose **D2** to L-glucose **L2** concentrations approaches 1 (highlighted in blue). Extrapolation of this data gives the glucose concentration in 50% diluted human serum as 2.85-2.95 mM. The concentration of D-glucose in the undiluted serum is thus determined as 5.7-5.9 mM.

[D2] / mM	ϑ_{260} / mdeg
0.00	-12.63
0.38	-9.06
0.96	-6.06
1.51	-3.81
2.01	-2.42
2.21	-1.67
2.30	-1.24
2.49	-0.94
2.71	-0.34
3.14	0.35
3.92	1.54
4.92	2.94
5.77	3.67

Titrations of D-Glucose into Receptor 1 + L-Glucose in Glucose-Free Human Serum (Calibrations for Single-Point Determinations).

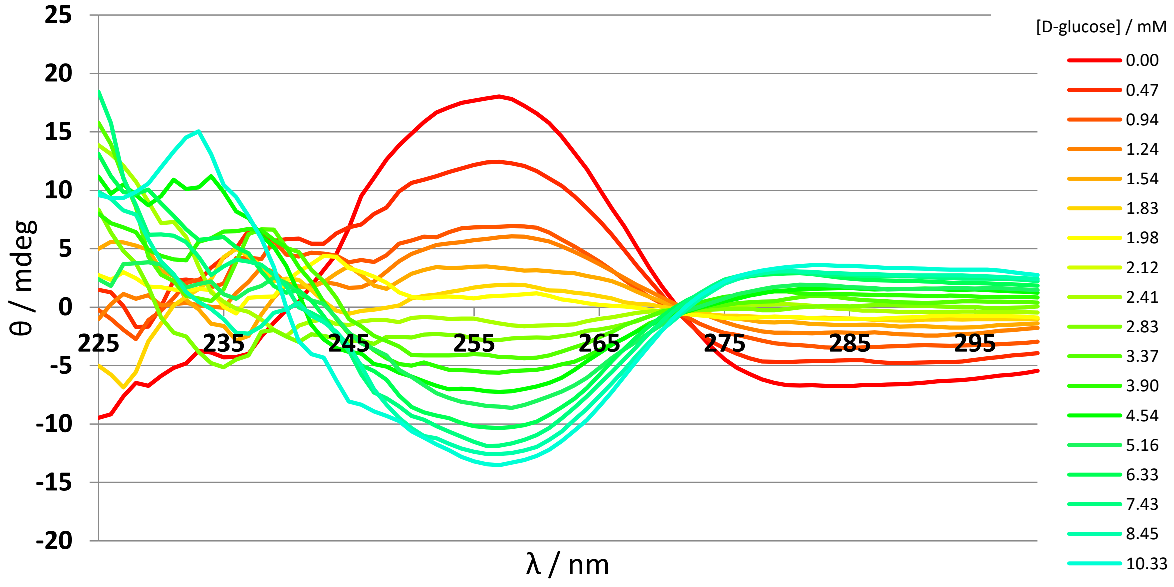

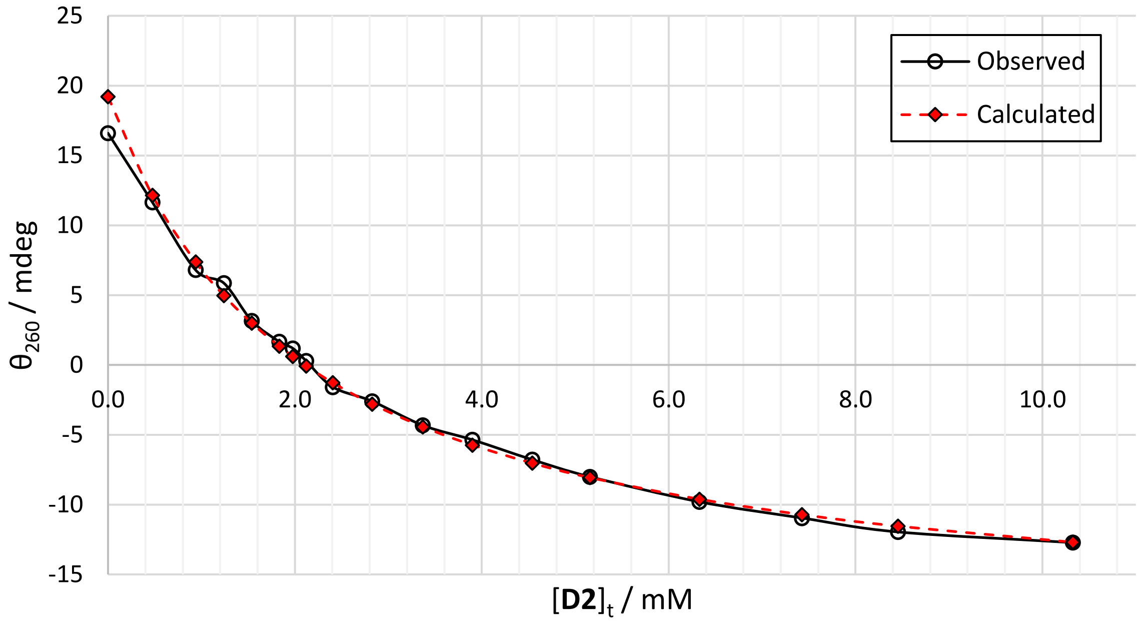

Figure S8 *[L-Glucose] = 2 mM.* Top: CD spectra for the titration of D-glucose **D2** (40 mM) into receptor **1** (0.25 mM) plus L-glucose **L2** (2 mM) in diluted glucose-free human serum (50% v/v) at 298 K. Bottom: The CD intensity at 260 nm (ϑ_{260}) plotted against increasing concentration of D-glucose (mM) (open circles) and fitted to equation (1) by variation of *k*. The best fit (red diamonds) was obtained for *k* = -76802 ± 2366.67 (3.08%) mdeg M^{-1}.

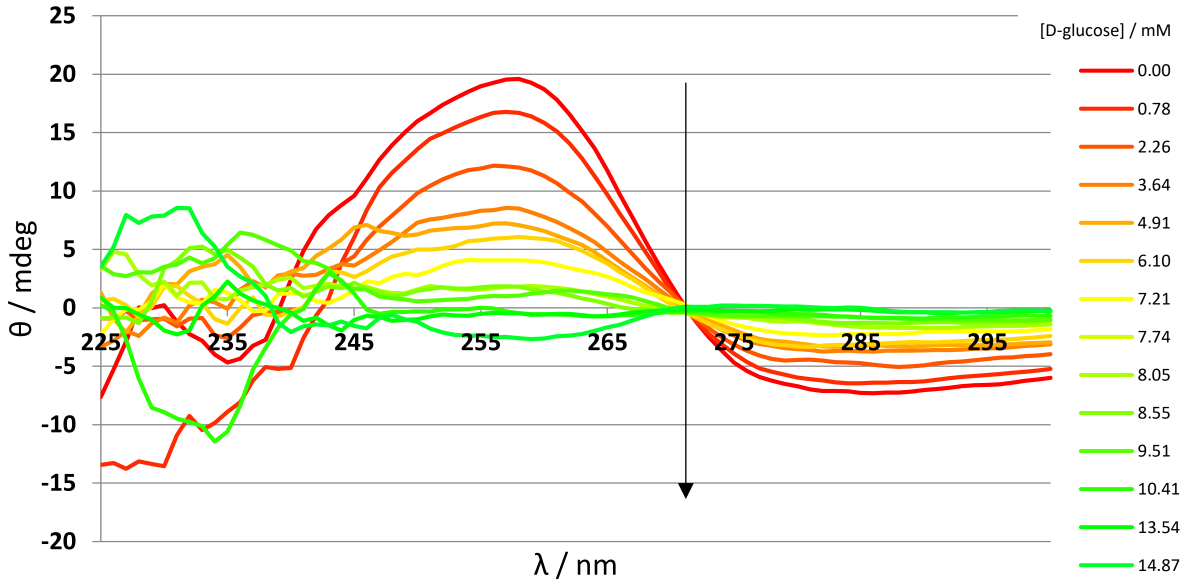

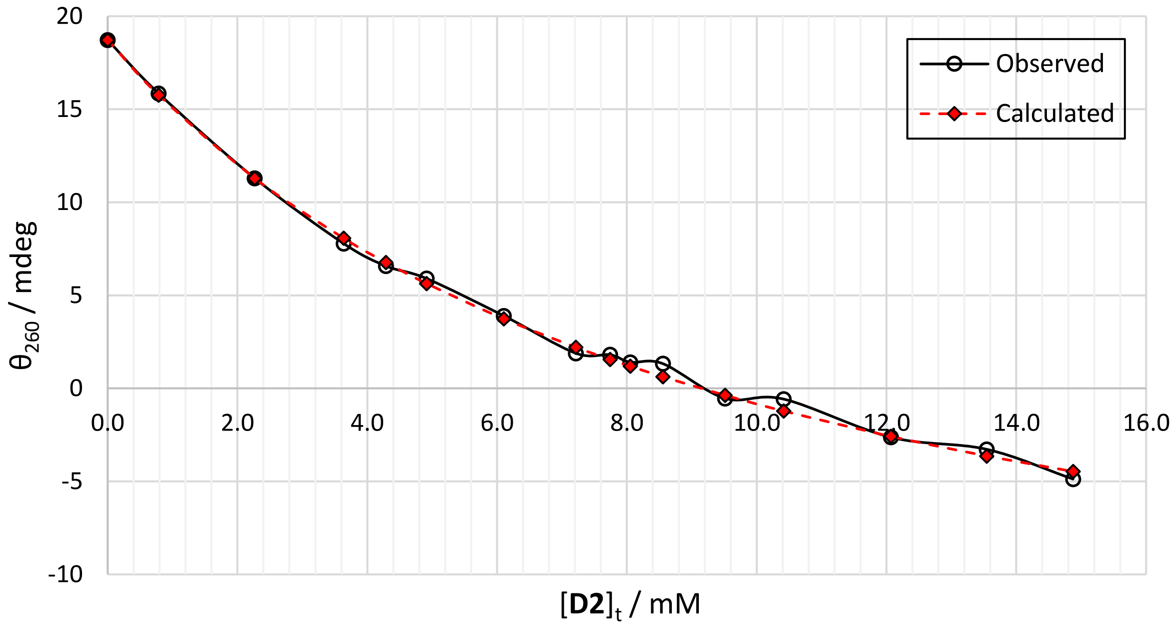

Figure S9 *[L-Glucose] = 8 mM.* Top: CD spectra for the titration of D-glucose **D2** (40 mM) into receptor **1** (0.25 mM) plus L-glucose **L2** (8 mM) in diluted glucose-free human serum (50% v/v) at 298 K. Bottom: The CD intensity at 260 nm (ϑ_{260}) plotted against increasing concentration of D-glucose (mM) (open circles) and fitted to equation (1) by variation of *k*. The best fit (red diamonds) was obtained for *k* = -74906 ± 1532.71 (2.05%) mdeg M^{-1}.

Single-Point Determination of D-Glucose in Filtered Human Serum

Filtered human serum was prepared as described earlier under "preparation of biological media". A sample (125 µL) was placed in a cuvette. To this was added a solution (125 µL) of receptor **1** (0.5 mM) L-glucose (4 or 16 mM) in phosphate buffer (pH 7.4, 20 mM), such that the final concentrations of added phosphate and **1** were 10 mM and 0.25 mM respectively, and the concentration of L-glucose was 2 or 8 mM. The cuvette was shaken and the CD spectrum recorded using the parameters employed for the calibration curves (Figures S8, S9). The CD intensity at 260 nm (ϑ_{260}) was used to calculate the concentration of D-glucose in the sample, through equation (S3), derived by rearrangement of equation (S2) as shown below. Equation (S3) is equivalent to equation (2) from the main paper.

$$\theta = k([\mathrm{HD}] - [\mathrm{HL}]) = k[\mathrm{H}]_t \frac{([\mathrm{D}]_t - [\mathrm{L}]_t)}{([\mathrm{L}]_t + [\mathrm{D}]_t)} \qquad \text{(S2)}$$

$$\theta([\mathrm{L}]_t + [\mathrm{D}]_t) = k[\mathrm{H}]_t([\mathrm{D}]_t - [\mathrm{L}]_t)$$

$$\theta[\mathrm{D}]_t - k[\mathrm{H}]_t[\mathrm{D}]_t = -k[\mathrm{H}]_t[\mathrm{L}]_t - \theta[\mathrm{L}]_t$$

$$[\mathrm{D}]_t(\theta - k[\mathrm{H}]_t) = -[\mathrm{L}]_t(k[\mathrm{H}]_t + \theta)$$

$$[\mathrm{D}]_t = -\frac{[\mathrm{L}]_t(k[\mathrm{H}]_t + \theta)}{k[\mathrm{H}]_t - \theta} \qquad \text{(S3)}$$

The concentration of D-glucose, $[\mathrm{D}]_t$, is thus available from the concentration of added L-glucose $[\mathrm{L}]_t$, the CD intensity, ϑ, the concentration of receptor, [H], and constant *k* from the calibration experiment. Because the procedure involves dilution of the sample by 50%, the value obtained must be doubled to give the concentration of D-glucose in the original sample.

Using the above procedure, six independent measurements were made on the human serum used in this work. The results are shown in Tables S3 ([**L2**] = 2 mM) and S4 ([**L2**] = 8 mM). Analysis of the serum by conventional glucose oxidase-based methodology, using a YSI 2300 STAT Plus analyser, yielded values of 5.65 and 5.90 mM (two independent measurements).

Table S3 Measured CD intensities of diluted filtered human serum (50% v/v) containing receptor **1** (0.25 mM) and L-glucose (2 mM), with D-glucose concentrations derived by application of equation (S3). The three independent values are averaged and doubled to give the D-glucose concentration in the undiluted serum.

	ϑ_{260} / mdeg	[D2] (diluted serum) / mM	[D2] (original serum) / mM
Run 1	-3.50	2.89	5.78
Run 2	-3.17	2.79	5.58
Run 3	-3.37	2.85	5.70
Average	-3.35	2.84	5.69

Table S4 Measured CD intensity of diluted filtered human serum (50% v/v) containing receptor **1** (0.25 mM) and L-glucose (8 mM), with D-glucose concentrations derived by application of equation (S3). The three independent values are averaged and doubled to give the D-glucose concentration in the undiluted serum.

	ϑ_{260} / mdeg	[D2] (diluted serum) / mM	[D2] (original serum) / mM
Run 1	8.68	2.94	5.87
Run 2	8.71	2.93	5.85
Run 3	8.93	2.84	5.68
Average	8.77	2.90	5.80

Titration of D-Glucose into Receptor 1 + L-Glucose in Glucose-Free Cell Culture Medium (Calibration for Single-Point Determinations).

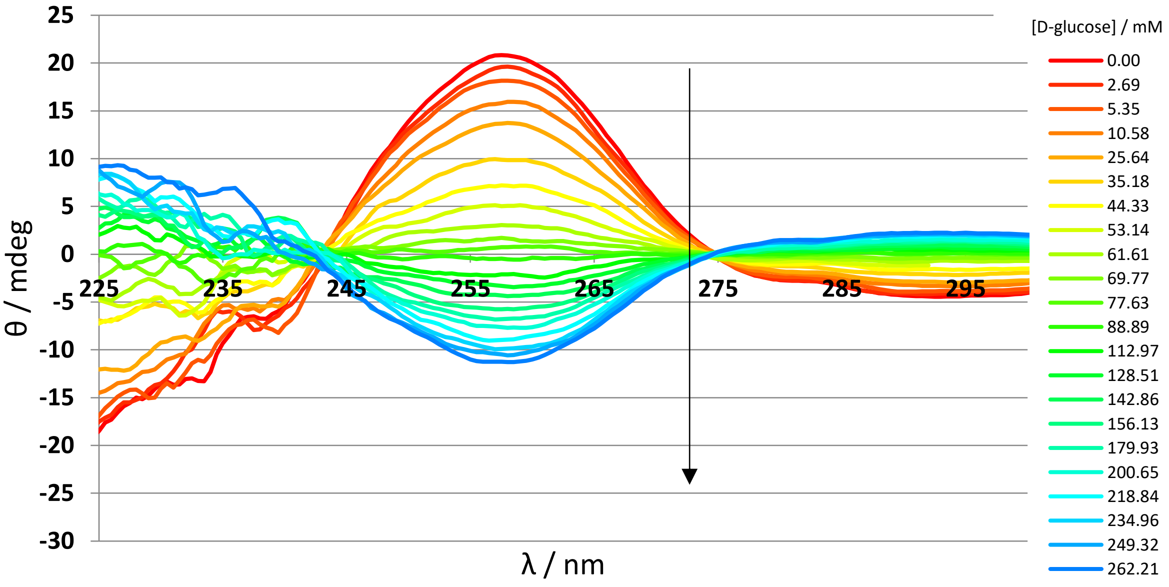

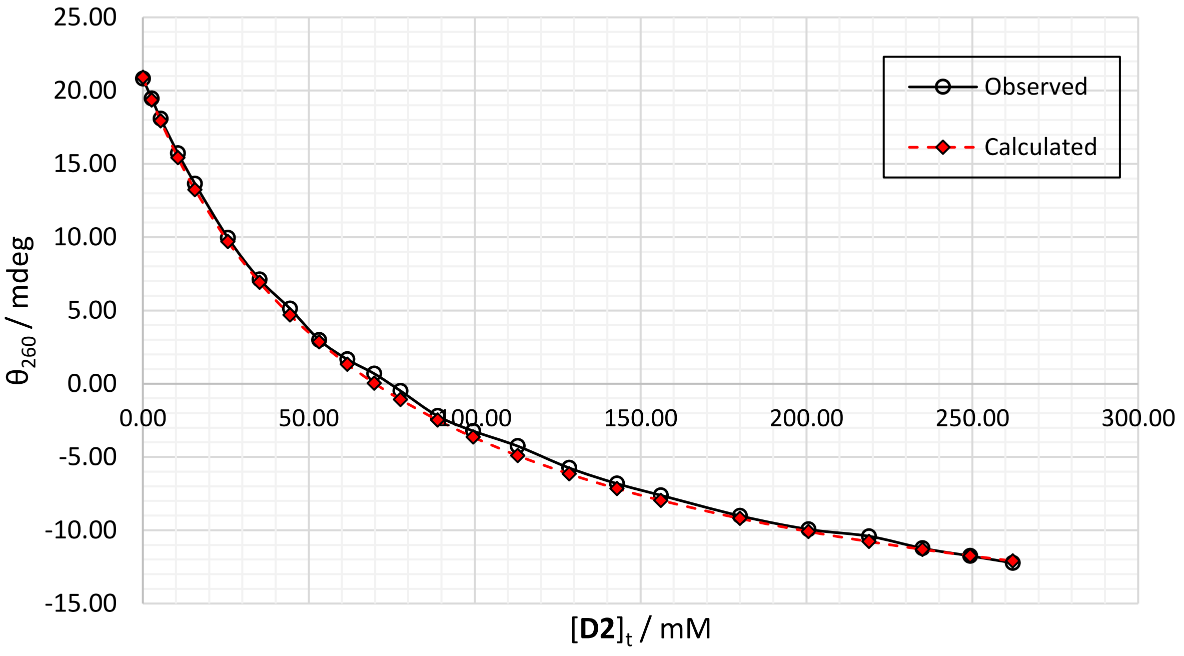

Figure S10. *[L-Glucose] = 50 mM.* Top: CD spectra for the titration of D-glucose **D2** (500 mM) into receptor **1** (0.25 mM) plus L-glucose **L2** (50 mM) in diluted glucose-free cell culture medium (50% v/v) at 298 K. Bottom: The CD intensity at 260 nm (ϑ_{260}) plotted against increasing concentration of D-glucose (mM) (open black circles) and fitted to equation (1) by variation of *k*. The best fit (red diamonds) was obtained for *k* = -83608 ± 1362.27 (1.63%) mdeg M^{-1}.

Single-Point Determination of D-Glucose in Glucose-Free Cell Culture Medium

D-Glucose was added to glucose-free cell culture medium, such that the final glucose concentration was 70 mM. A sample (125 µL) was placed in a cuvette. To this was added a solution (125 µL) of receptor **1** (1.6 mM), L-glucose (625 mM) and phosphate buffer (pH 7.4, 100 mM), such that the final concentrations of added phosphate and receptor **1** were 10 mM and 0.25 mM respectively, and the concentration of L-glucose was 50 mM. The cuvette was shaken and the CD spectrum was recorded using the parameters employed for the calibration curve (Figure S10). The CD intensity at 260 nm (ϑ_{260}) was used to calculate the concentration of D-glucose in the sample, through equation (S3), derived by rearrangement of equation (S2) as shown above. Equation (S3) is equivalent to equation (2) from the main paper.

Table S5 Measured CD intensities of diluted D-glucose–spiked cell culture medium (50% v/v) containing receptor **1** (0.25 mM) and L-glucose (50 mM), with D-glucose concentrations derived by application of equation (S3). The two independent values are averaged and doubled to give the D-glucose concentration in the undiluted cell culture medium.

	ϑ_{260} / mdeg	[D2] (diluted cell culture medium) / mM	[D2] (original serum) / mM
Run 1	7.32	33.7	67.4
Run2	6.98	34.9	69.8
Average	7.15	34.3	68.6

Titration of D-Glucose into Receptor 1 + L-Glucose in Glucose-Free Peroni Beer (Calibration for Single-Point Determinations).

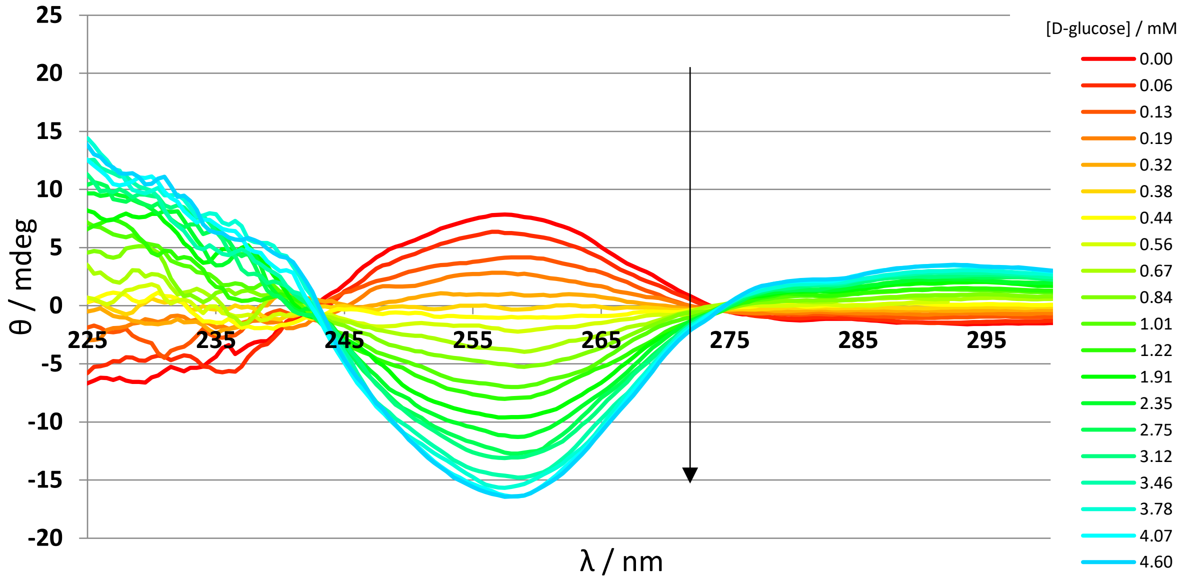

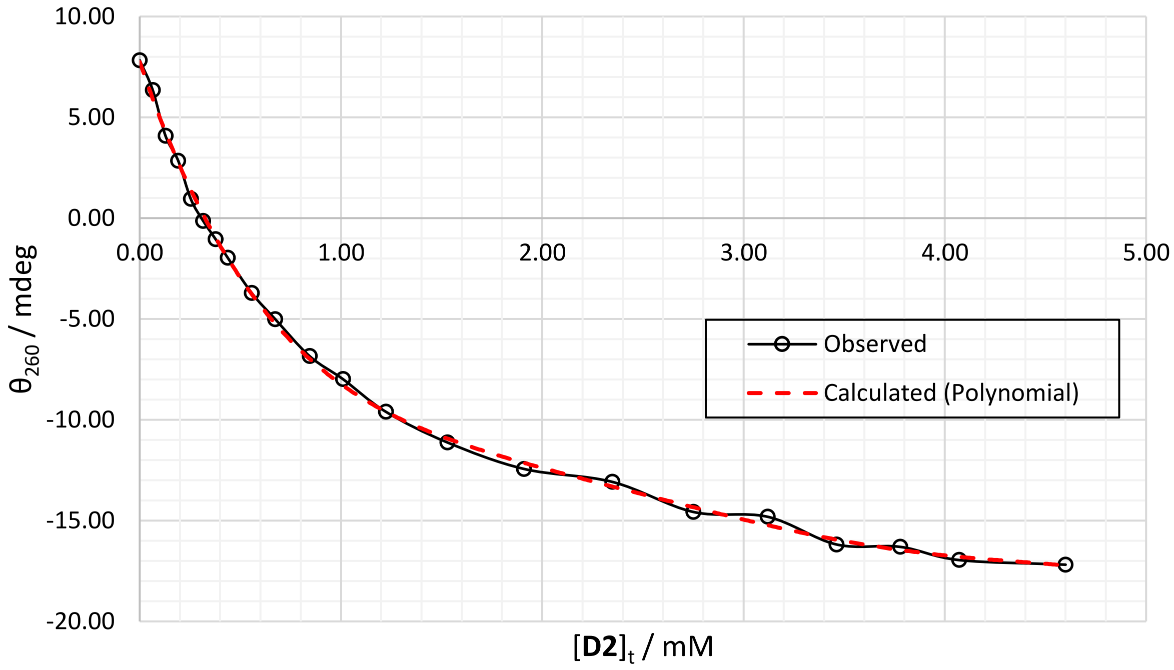

Figure S11. *[L-Glucose] = 0.2 mM.* Top: CD spectra for the titration of D-glucose **D2** (12 mM) into receptor **1** (0.25 mM) plus L-glucose **L2** (0.2 mM) in diluted glucose-free Peroni beer (50% v/v) at 298 K. Bottom: The CD intensity at 260 nm (ϑ_{260}) plotted against increasing concentration of D-glucose (mM) (open circles) and fitted to a polynomial equation (S4, generated in Excel): ϑ_{260} = -0.0724[**D2**]$_t^5$ + 1.1163[**D2**]$_t^4$ - 6.5896[**D2**]$_t^3$ + 18.951[**D2**]$_t^2$ - 29.383[**D2**]$_t$ + 7.7475, R^2 = 0.9991.

Single-Point Determination of D-Glucose in Peroni Beer

Glucose-free Peroni beer was prepared as described earlier. D-glucose was added, such that the final glucose concentration was 400 µM. A sample (125 µL) was placed in a cuvette. To this was added a solution (125 µL) of receptor **1** (1.6 mM), L-glucose (2.5 mM) and phosphate buffer (pH 7.4, 100 mM), such that the final concentrations of added phosphate and **1** were 10 mM and 0.25 mM respectively, and the concentration of L-glucose was 0.2 mM. The cuvette was shaken and the CD spectrum was recorded using the parameters employed for the calibration curve (Figure S11). The CD intensity at 260 nm (ϑ_{260}) was used to calculate the concentration of D-glucose in the sample by inserting the value into equation (S4) and solving for [**D2**].‡

Table S6 Measured CD intensities of diluted D-glucose–spiked Peroni beer (50% v/v) containing receptor **1** (0.25 mM) and L-glucose (200 µM), with D-glucose concentrations derived by application of equation (S4). The two independent values are averaged and doubled to give the D-glucose concentration in the undiluted beer.

	ϑ_{260} / mdeg	[D2] (diluted beer) / µM	[D2] (original beer) / µM
Run 1	2.52	203	406
Run 2	2.46	205	410
Average	2.49	204	408

‡ Solutions were obtained using the mathematical website https://www.wolframalpha.com.
